# Increased difference between slow and forced vital capacity is associated with reduced exercise tolerance in COPD patients

**DOI:** 10.1186/1471-2466-14-16

**Published:** 2014-02-07

**Authors:** Wei Yuan, Xin He, Qiu-Fen Xu, Hao-Yan Wang, Richard Casaburi

**Affiliations:** 1 Department of Respiratory Medicine, Beijing Friendship Hospital, Capital Medical University, No. 95 Yong An Road, Xichen District, Beijing 100050, China; 2 Rehabilitation Clinical Trials Center, Los Angeles Biomedical Research Institute at Harbor-UCLA Medical Center, Los Angeles, USA

**Keywords:** Chronic obstructive pulmonary disease, Spirometry, Exercise

## Abstract

**Background:**

A higher slow vital capacity (VC) compared with forced vital capacity (FVC) indicates small airway collapse and air trapping. We hypothesized that a larger difference between VC and FVC (VC-FVC) would predict impaired exercise capacity in patients with chronic obstructive pulmonary disease (COPD).

**Methods:**

Pulmonary function and incremental cardiopulmonary exercise responses were assessed in 97 COPD patients. Patients were then divided into two groups: one in which VC > FVC (n = 77) and the other in which VC ≤ FVC (n = 20).

**Results:**

Patients with VC > FVC had lower FEV_1_ and peak oxygen uptake (VO_2_/kg) compared with patients with VC ≤ FVC. There was a significant inverse correlation for the entire group between VC-FVC and peak VO_2_/kg (r = -0.404; p < 0.001). There was also a direct correlation between FEV_1_% pred and peak VO_2_/kg (r = 0.418; p < 0.001). The results of the multivariate regression analysis with peak VO_2_/kg as the dependent variable showed that VC-FVC, FEV_1_(% pred) and age were all significant independent predictors of peak VO_2_/kg. The model explained 35.9% of the peak VO_2_/kg variance.

**Conclusions:**

The difference between VC and FVC, easily measured by spirometry, can be used not only as an index of severity of airflow limitation, but also to predict exercise performance in COPD patients.

## Backgrounds

Persistent and progressive airflow limitation is a main characteristic of chronic obstructive pulmonary disease (COPD) [1]. As a result, patients with COPD experience dyspnea and impaired exercise capacity that progress over time. The disease severity has usually been graded based on the degree of airflow limitation, as measured by a reduced forced expiratory volume in one second (FEV_1_). However, there is only a weak correlation between FEV_1_, symptoms and impairment of a patient’s health-related quality of life [1]. The long-term rate of deterioration in exercise capacity in patients with COPD was found to be more rapid than the rate of decline in FEV_1_. The change in peak oxygen uptake (VO_2_) over time was only weakly correlated with the change in FEV_1_[2]. On the other hand, measurements of lung volumes, such as the inspiratory capacity (IC), correlate better with patient functional capabilities than do measurements of FEV_1_[3-5].

IC has been wildly used to study pulmonary hyperinflation because it can be simply measured by spirometry. A low resting IC reflects pulmonary hyperinflation, which is defined as an abnormally increased lung volume at the end of tidal expiration and is present in moderate to severe COPD due to expiratory flow limitation and destructive changes of emphysema. Serial IC measurements have be used to track dynamic hyperinflation (DH) during exercise, which occurs when ventilatory demand increases, leaving less time for expiration and resulting in air trapping within the lungs [6-8]. Several studies have showed that reduction in resting IC or dynamic IC constrains tidal volume expansion during exercise and contributes importantly to reduced exercise performance in patients with COPD [6,7,9,10].

Vital capacity, simply measured by spirometry, is a lung volume measurement that can be measured as slow vital capacity (VC) or forced vital capacity (FVC). There is little or no difference between VC and FVC (VC-FVC) in normal subjects [11]. However, some studies have found that FVC is smaller than VC both in asthma patients and COPD patients and the difference between the two parameters is related to airflow limitation, small airway collapse and gas trapping [11-13].

We hypothesized that COPD patients with larger difference between VC and FVC would have lower exercise capacity. To test this hypothesis, we performed spirometry and cardiopulmonary exercise testing and examined the relationship of the difference between VC and FVC to exercise performance in patients with COPD.

## Methods

### Subjects

We recruited 97 patients with COPD who satisfied the following criteria: FEV_1_/FVC ratio < 0.7 and FEV_1_ of 30 to 80% predicted after inhalation of 400 ug albuterol [1] and in a stable condition for at least 6 weeks. Patients with diagnoses of interstitial lung disease or asthma were excluded. All patients received written and verbal information about the exercise testing. The study was approved by the Ethics Committee of Beijing Friendship Hospital.

### Pulmonary function tests

Spirometry, including VC, FVC and FEV_1_ was performed using MasterScreen system (MasterScreen Body, CareFusion, Hoechberg, Germany) before exercise testing in all subjects. Procedures were carried out according to ATS/ERS standards [14]. VC was obtained by asking subjects to take in a full inspiration and then breathe out to the limit of full expiration in a relaxed manner except near end-inspiration and end-expiration. VC maneuvers was performed before FVC maneuvers, each of which was performed 3 times, with the highest value of each selected.

### Exercise testing

Patients performed a progressive incremental exercise testing to a symptom-limited maximum on an electronically braked cycle ergometer (ViaSprint, CareFusion, Hoechberg, Germany). The protocol consisted of 3 minutes of rest, 3 minutes of unloaded pedaling, and minute-by-minute increments at a work rate of 5 to 20 W/min. Minute ventilation, oxygen uptake (VO_2_), and carbon dioxide output (VCO_2_) were acquired breath-by-breath using a computerized system (OxyconDelta, CareFusion, Hoechberg, Germany). Maximal heart rate (HR) predicted for age was calculated as 220-age [15].

### Statistical analysis

Data with normal distribution are presented as means ± SD and data not normally distributed are presented as median and interquartile range. Patients were classified into two groups using the difference between VC and FVC: one group with VC > FVC and the other group with VC ≤ FVC. Differences between the two groups were analyzed using independent sample t-tests. Comparison between the two groups in VC-FVC was performed using the Mann-Whitney U-test. Correlations between lung function and exercise variables were assessed by Pearson’s correlation coefficient. A multivariate linear regression analysis was used to determine the independent association of lung function and other variables with exercise capacity. A *p*-value of less than 0.05 was considered statistically significant.

## Results

Ninety seven patients with COPD were involved in this study. Of these, 4 patients were staged as mildly impaired (spirometry stage 1), 35 patients were moderately impaired (stage 2), 49 patients were severely impaired (stage 3), and 9 patients were very severely impaired (stage 4) according to GOLD classification [1]. The descriptive characteristics of the subjects are summarized in Table 1.

**Table 1:** Patient characteristics

**Characteristics**	**Data**
Male	97
Age, yr	64±8
Weight, kg	70.37±10.68
VC, L	2.72±0.72
FVC, L	2.56±0.74
VC-FVC, † L	0.11 (0.02-0.24)
FEV_1_, L	1.31±0.54
FEV_1_, % predicted	48.85±18.13
FEV_1_/FVC, %	50.44±10.69
Peak VO_2_, ml/min/kg	14.45±4.92
Peak work rate, watts	71.98±34.98
Peak HR, beats/min	128±16

Data with normal distribution are presented as mean ± SD.

†: Expressed as median (interquartile range).

VC = vital capacity; FVC = forced vital capacity; FEV_1_ = forced expiratory volume in one second; VC-FVC = difference between VC and FVC; VO_2_ = oxygen uptake; HR = heart rate.

**Figure 1:**
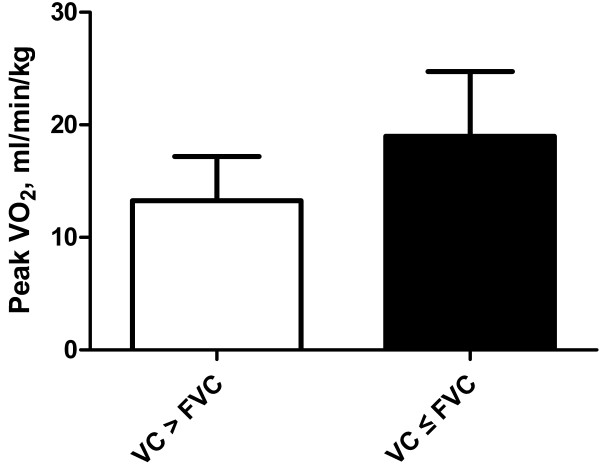
**Peak VO**_**2**_**/kg in COPD patients with VC > FVC and VC ≤ FVC.** Patients with VC > FVC had a lower peak VO_2_/kg compared with patients with VC ≤ FVC (p < 0.001).

The patients were divided into those with VC > FVC (n = 77) and those with VC ≤ FVC (n = 20). The lung function and exercise parameters of these two groups are shown in Table 2. The mean age was similar in the two groups. Patients with VC > FVC had lower FEV_1_ and peak VO_2_/kg compared with patients with VC ≤ FVC (Figure 1). The peak work rate reached in the group of patients with VC > FVC was lower than in the patients with VC ≤ FVC, but without reaching statistical significance. There was no significant difference in heart rate at peak exercise between the two groups.

**Table 2:** Lung function and exercise responses of patients with COPD classified by difference between VC and FVC

	**VC > FVC**	**VC ≤ FVC**	**p-value**
Patients, n	77	20	
Age, yr	65±8.2	62±7.7	0.187
VC-FVC, † L	0.16 (0.07-0.27)	-0.20 (-0.30--0.03)	<0.001
FEV_1_, L	1.21±0.46	1.67±0.68	0.008
FEV_1_, % predicted	46.41±16.13	58.26±22.44	0.009
FEV_1_/FVC, %	49.29±10.49	54.89±10.54	0.036
Peak VO_2_, ml/min/kg	13.26±3.92	18.99±5.75	<0.001
Peak work rate, watts	68.53±34.45	85.10±34.74	0.059
Peak HR, beats/min	128±16	128±11	0.983

Data with normal distribution are presented as mean ± SD.

†: Expressed as median (interquartile range).

VC = vital capacity; FVC = forced vital capacity; FEV_1_ = forced expiratory volume in one second; VC-FVC = difference between VC and FVC; VO_2_ = oxygen uptake; HR = heart rate.

**Figure 2:**
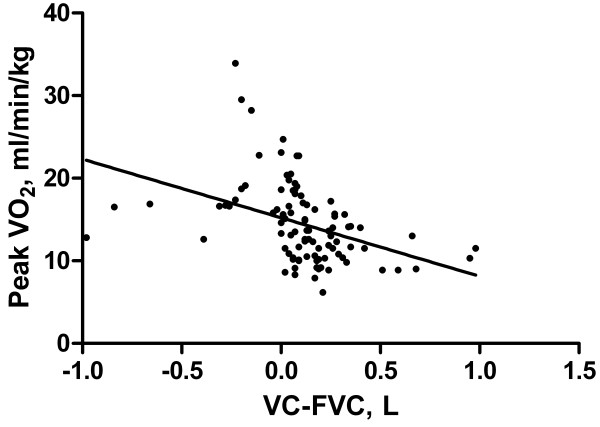
**Relationship between VC-FVC and peak VO**_**2**_**/kg.** There was a significant inverse correlation for the entire group between VC-FVC and peak VO_2_/kg (r = -0.404; p < 0.001).

**Figure 3:**
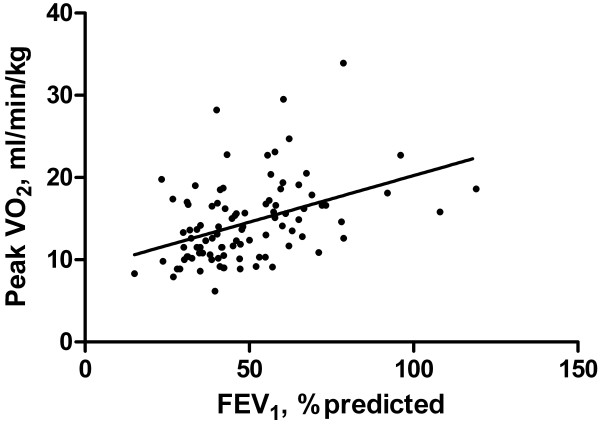
**Relationship between FEV**_**1**_**, % predicted and peak VO**_**2**_**/kg.** There was a direct correlation between FEV_1_ % pred and peak VO_2_/kg (r = 0.418; p < 0.001).

There was a significant inverse correlation for the entire group between VC-FVC and peak VO_2_/kg (r = -0.404; p < 0.001) (Figure 2). There was also a direct correlation between FEV_1_ % pred and peak VO_2_/kg (r = 0.418; p < 0.001) (Figure 3).

The results of the multivariate regression analysis with peak VO_2_/kg as the dependent variable is presented in Table 3. Difference between VC and FVC, FEV_1_% pred and age were all significant and independent predictors of peak VO_2_/kg. The model explains 35.9% of the peak VO_2_ variance.

**Table 3:** **Multivariate regression analysis in COPD patients with VO**_**2 **_**(in ml/min/kg) at peak exercise as the dependent variable**

**Independent variable**	**Coefficient**	**p-value**
Age, yr	-0.173	0.001
FEV_1_, % predicted	0.098	<0.001
VC-FVC, L	-5.122	0.001

VC-FVC = difference between VC and FVC; FEV_1_ = forced expiratory volume in one second.

## Discussion

In this study, patients with VC > FVC had a lower FEV_1_ and a lower peak VO_2_/kg, compared with those with VC ≤ FVC. There was a significant and inverse correlation for the entire group between VC-FVC and peak VO_2_/kg. There was also a direct correlation between FEV_1_ % pred and peak VO_2_/kg. The results of the multivariate regression analysis show that difference between VC and FVC, FEV_1_ % pred and age were significantly independent predictors of peak VO_2_/kg. These results support our hypothesis that a larger difference between VC and FVC is associated with reduced exercise capacity in patients with COPD.

FVC is defined as the volume of air exhaled with maximal forced effort from a maximal inspiration. VC is similar to the FVC, but the maneuver is not forced and it is performed in a relaxed manner, except near the end-inspiration and end-expiration. Several studies have explored differences between VC and FVC and their relationship to small airway function. Cohen et al. [16] evaluated the FVC/VC ratio in patients with bronchiolitis obliterans syndrome occurring after lung transplantation, in which small airways become progressively obliterated, leading to airway obstruction. They found that FVC/VC ratio decreased significantly with progressing bronchiolitis obliterans stages, which occurred independently of changes in FEV_1_. In asthma patients, it has been described that the difference between VC and FVC increases as the degree of obstruction increases. The difference can be used as an indicator of air trapping [11,17]. Similar results were found in the present study. We showed that FEV_1_% predicted was lower in patients with VC > FVC than in those with VC ≤ FVC, suggesting that difference between VC and FVC was significantly associated with the degree of airflow obstruction. There were 20 patients with VC ≤ FVC. The underlying mechanism for larger FVC than VC remains uncertain.

Although VC and FVC are often measured in lung function laboratory, the difference between them has less been evaluated. To our knowledge, there have been no previous studies describing the relationship between the difference (VC-FVC) and exercise capacity in patients with COPD. The current study explored this relationship and showed that there was a significant and inverse correlation between VC-FVC and peak VO_2_/kg (r = -0.404; p < 0.001). Combined with FEV_1_% predicted and age, the difference between VC and FVC could significantly account for 35.9% of the peak VO_2_/kg variance. This finding has an important implication for clinical practice. The difference between VC and FVC, simply measured by spirometry, can be used to predict exercise capacity in patients with COPD.

We may speculate on the underlying mechanism of association between the difference (VC-FVC) and exercise capacity. The first issue to consider is why a difference between VC and FVC exists in COPD. This has been explained by the following mechanism. Small airways, defined as those smaller than 2 mm in diameter, have no cartilaginous support and are subject to collapse when compressed [18]. During a slow VC maneuver, less thoracic gas compression occurs and greater air volume can be expired. In contrast, during an FVC maneuver, greater airway compression occurs and a smaller volume is expired. However, in healthy people this tendency is partly opposed by the attachment of the alveolar septa to the airway walls. In those with COPD, the airways tend to collapse during a forced expiration due to the reduction of alveolar attachments and airway abnormalities. Therefore, finding that VC is higher than FVC suggests small airway collapse and air trapping [17]. It further may indicate that static pulmonary hyperinflation is present. Since pulmonary hyperinflation constrains tidal volume expansion during exercise and contributes importantly to reduced exercise performance in patients with COPD, this seems a likely explanation for the association of the difference between VC and FVC and reduced exercise capacity in patients with COPD.

A limitation of the present study is that we did not measure inspiratory capacity during exercise, so as to better quantitate dynamic hyperinflation and more precisely reveal the mechanisms of association between VC-FVC and exercise performance in patients with COPD. This should be addressed in future studies.

## Conclusion

We have studied the association between VC, FVC and exercise capacity in patients with COPD. We found that the difference between VC and FVC at rest, which is easily obtained from spirometric results and does not depend on predicted population values, can be used not only as an index of severity of airflow limitation, but also to predict exercise performance in patients with COPD.

## Abbreviations

COPD: Chronic obstructive pulmonary disease; VC: Slow vital capacity; FVC: Forced vital capacity; VC-FVC: Difference between VC and FVC; FEV1: Forced expiratory volume in one second; VO2: Oxygen uptake; IC: Inspiratory capacity.

## Competing interests

The authors declare that they have no competing interests.

## Authors’ contributions

Conceptualization: HYW. Study design: HYW; QFX. Study implementation: WY; XH; QFX. Data analysis: HYW; WY. Manuscript preparation: HYW; RC. All authors read and approved the final manuscript.

## Pre-publication history

The pre-publication history for this paper can be accessed here:

http://www.biomedcentral.com/1471-2466/14/16/prepub
